# Serial assessment of left atrial deformation in patients undergoing pulmonary vein isolation: a cardiovascular magnetic resonance feasibility study

**DOI:** 10.1186/1532-429X-18-S1-P362

**Published:** 2016-01-27

**Authors:** Leonard Bergau, Tobias Tichelbaecker, Johannes T Kowallick, Lars Lüthje, Thomas H Fischer, Christina Unterberg-Buchwald, Joachim Lotz, Markus Zabel, Gerd Hasenfuss, Wieland Staab, Andreas Schuster

**Affiliations:** 1 grid.411984.10000000104825331Cardiology and Pneumology, University Medical Center Goettingen, Goettingen, Germany; 2 grid.411984.10000000104825331Institute for Diagnostic and Interventional Radiology, University Medical Center Goettingen, Goettingen, Germany

## Background

Left atrial (LA) performance quantification using Cardiovascular Magnetic Resonance (CMR) is of growing interest. There is evidence that declined left atrial function is associated with a poorer outcome following pulmonary vein isolation (PVI) in atrial fibrillation (AF). Furthermore, the influence of PVI on left atrial performance has not yet been investigated comprehensively. This study was designed to investigate the feasibility of CMR using comprehensive myocardial feature tracking (CMR-FT) and volumetric analyses for serial investigation of atrial performance before and after PVI.

## Methods

Eight consecutive patients (age 59 ± 11 years, 50 % male, 38 % persistent AF) undergoing PVI was included. All patients received manually guided PVI using open-irrigated radiofrequency ablation catheters. All patients underwent CMR (either 1.5 or 3T) ahead of PVI and 3 months afterwards. LA longitudinal strain and strain rate (SR) parameters as well as fractionated LA-volume changes were derived from long-axis 2- and 4-chamber cine images using dedicated software (2D CPA MR, TomTec, Germany and QMass Version 7.6, Medis Medical Systems, The Netherlands). LA performance was assessed calculating LA reservoir function (total strain [ε_s_], peak positive SR [SRs]), LA conduit function (passive strain [ε_e_], peak early negative SR [SRe]) and LA booster pump function (active strain [ε_a_], late peak negative SR [SRa]).

## Results

**Figure 1:**
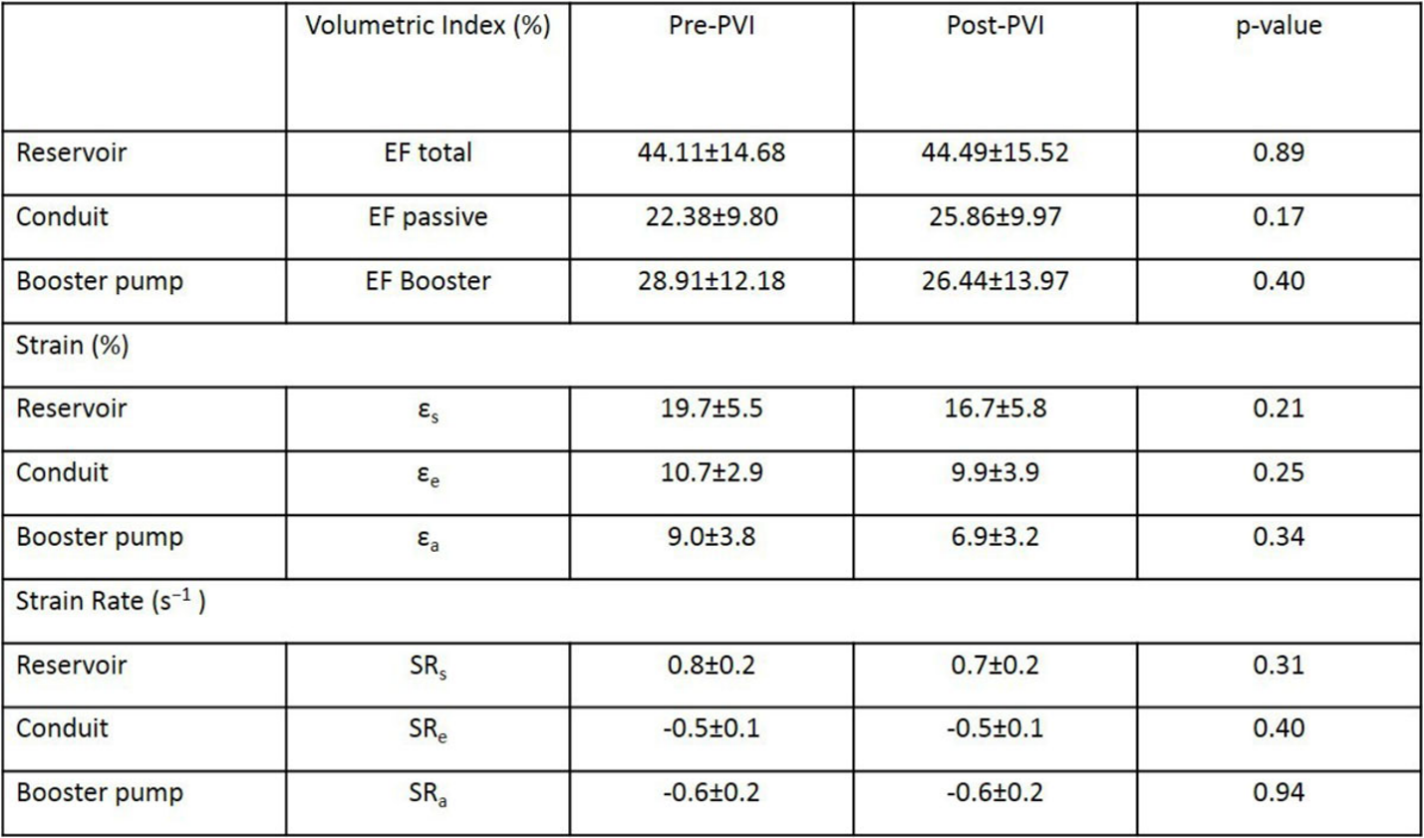
Figure 1

CMR was obtained in stable sinus rhythm in all studies. CMR-FT atrial performance analysis was feasible in all patients. There was no statistical significant difference in atrial phasic performance based on volumes, strain and strain rate before and after PVI (see Figure 1). However, deterioration of left atrial performance appeared to be more likely in patients with repeated ablations, whereas atrial performance following a single PVI remained stable.

## Conclusions

Serial assessments of atrial phasic performance using CMR is feasible before and after PVI. Future studies will need to relate changes in these novel quantitative parameters to atrial fibrosis and outcome to define their incremental clinical merit.

